# The Overexpression of *NUC* Promotes Development and Increases Resistance to Nitrogen Deficiency in *Arabidopsis thaliana*

**DOI:** 10.3390/ijms222111413

**Published:** 2021-10-22

**Authors:** Jing Ling, Xing Huang, Yanxia Jia, Weiqi Li, Xudong Zhang

**Affiliations:** 1 Germplasm Bank of Wild Species, Kunming Institute of Botany, Chinese Academy of Sciences, Kunming 650201, China; lingjing@mail.kib.ac.cn (J.L.); huangxing@mail.kib.ac.cn (X.H.); jiayanxia@mail.kib.ac.cn (Y.J.); 2 University of the Chinese Academy of Sciences, Beijing 100049, China

**Keywords:** NUTCRACKER, development, primary roots, lateral roots, nitrogen response gene, nitrogen deficiency

## Abstract

NUTCRACKER (NUC) is a transcription factor expressed in multiple tissues, but little is known about its physiological roles. In this study, we explored the physiological function of *NUC* with the *Arabidopsis* knockout, rescue, and overexpression lines. We found that *NUC* overexpression promoted development at the germination, seedling, and juvenile stages. *NUC* overexpression increased resistance to nitrogen (N) deficiency stress by increasing the chlorophyll content, suppressing anthocyanin accumulation, and increasing the biomass under N deficiency. In contrast, the absence of *NUC* did not affect such characteristics. N deficiency significantly increased the expression of *NUC* in leaves but did not affect the expression of *NUC* in roots. The overexpression of *NUC* promoted primary root length under both normal and N deficiency conditions. Furthermore, we found that the N-responsive and lateral-root-related genes *TGA1* and *NRT2.4* had NUC-binding sites in their promoter regions and that their expression was upregulated by *NUC* under N deficiency. The overexpression of the *NUC* increased the number and length of the lateral roots under N deficiency through inducible promotion. Multiple lines of investigation suggest that the regulatory function of the NUC could be bypassed through its redundant MAGPIE (MGP) when the NUC is absent. Our findings provide novel insight into NUC’s functions and will assist efforts to improve plants’ development and resistance to nutrient stresses.

## 1. Introduction

NUTCRACKER (NUC/IDD8, AT5G44160), which belongs to the INDETERMINATE DOMAIN (IDD) family, is a Cys2His2 zinc-finger domain (C2H2) transcription factor. NUC is expressed in many plant tissues but is especially common in vegetative tissues [1]. In *Arabidopsis*, NUC participates in sugar metabolism, affecting the expression of sucrose transporter genes and sucrose synthase genes and contributing to flowering time [1]. NUC can be phosphorylated by AKIN10, which reduces the transcriptional activity of NUC [2]. With its homologous proteins JACKDAW (JKD) and MAGPIE (MGP), NUC was previously described as a direct SCARECROW (SCR) and SHORT ROOT (SHR) transcriptional target, and it regulates periclinal divisions in ground tissues [3,4]. We previously found that it can regulate root tip development in *A. thaliana* [5]. To the best of our knowledge, only two cases of its physiological roles, positively regulating flowering and root development, have been reported thus far. Whether NUC has other roles remains unknown.

Nitrogen (N) is essential for plant growth and development [6]. The availability of N resources in soil is very limited, and N fertilizers are routinely used to increase agricultural productivity. However, owing to the low nitrogen use efficiency (NUE) of plants, a large portion of N fertilizers cannot be absorbed, which leads to considerable financial costs and many environmental problems, such as eutrophication and soil acidification [7]. Understanding how plants respond to the changing levels and forms of N available in the rhizosphere, as well as improving the NUE of plants, are necessary for solving these problems and improving the sustainability of the agricultural ecosystem.

In order to adapt to fluctuations in the availability of N in nature, plants have evolved sophisticated mechanisms for improving their NUE. The nitrogen responses of plants can be divided into short- and long-term responses. Plants can sense N deficiency and rapidly regulate the expression of more than 1000 genes within a short period [8,9,10]. In long-term responses, plants usually regulate their root and shoot morphology, flowering time, and seed maturation and dormancy [11,12,13,14,15,16,17]. Nutrient capture is highly dependent on the architecture of the root system. Root systems are modified in order to better forage for N under N-deficient conditions through the addition of long primary roots, branching, and long lateral roots [18,19,20].

Previous studies have discovered many nitrogen-related transcription factors involved in root development [21,22]. For example, ANR1, identified as the first transcription factor of the nitrate response, can regulate lateral root development [23,24]. TCP20 not only binds to the promoters of some N metabolism genes directly but also interacts with NLP6/7 to enhance lateral root growth [25,26]. TGA1 and TGA4 belong to the bZIP family and can regulate the expression of nitrate transporters and root development [27,28,29,30]. LBD37, LBD38, and LBD39 act as negative regulators of anthocyanin biosynthesis and negatively regulate the expression of N metabolism genes to regulate root development under nitrate-deficient conditions [31].

In the present study, we explored the function of NUC in the *Arabidopsis* knockout, rescue, and overexpression lines. We found that NUC had a positive role in the development and resistance of *Arabidopsis* to N deficiency stress. The overexpression of *NUC* under N deficiency promoted its development in multiple stages, constitutively promoting the length of the primary roots, and increased the number and length of the lateral roots through inducible promotion. The resistance of plants to N deficiency in *NUC* overexpression lines might result mainly from the regulation of *TGA1* and *NRT2.4* expression. These novel findings expand our understanding of the functions of *NUC* and will assist efforts to improve plant development, the promotion of NUE, and plants’ resistance to nutrient stresses.

## 2. Results and Discussion

### 2.1. The Overexpression of NUC Promotes Growth in Arabidopsis

**Figure 1:**
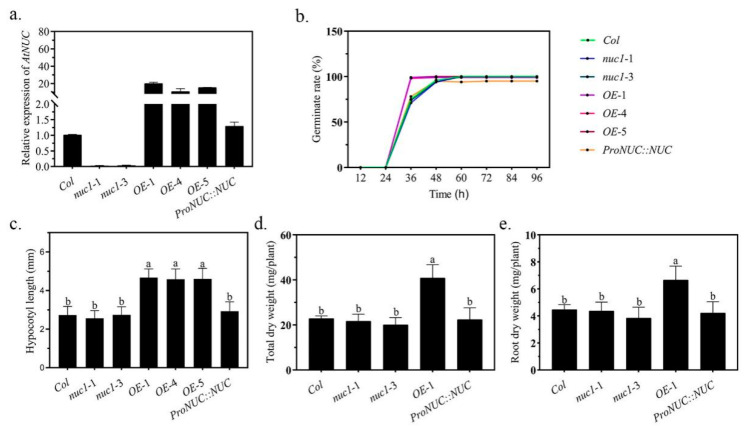
The effects of NUC on the development and growth of *Arabidopsis*. (**a**): Expression levels of *AtNUC* were measured by qRT–PCR in different genotype lines. Gene expression in *Col* was set to 1.0. The *Arabidopsis* tubulin gene was used as an external reference. Values are the mean ± SDs (*n* = 3). (**b**): Germination rate of different genotype lines grown on normal MS medium with a density of 160 plants per plate, 40 seedlings per line. (**c**): Hypocotyl length of 15-day-old seedlings grown on normal 1/2 strength MS medium. Values are the mean ± SDs (*n* ≥ 25). (**d**,**e**): Dry weights of whole plants and hydroponically cultured roots grown for 45 days, respectively. Values are the mean ± SDs (*n* ≥ 5). Different letters indicate significant difference between treatments according to Duncan’s multiple range test at *p* < 0.05.

We employed two T-DNA insertion lines, one recovery line, and three overexpression lines to test the potential functions of the NUC in the development and growth of plants (Figure 1a and Figure S1a). The *NUC* expression in two T-DNA insertion lines, *nuc1*-1 and *nuc1*-3, was blocked; in the recovery line, *ProNUC::NUC*, the expression was similar to that in *Col*; and in the overexpression lines, *OE*-1, *OE*-4, and *OE*-5, the expression was 20-, 10-, and 15-fold higher than in *Col*, respectively. We found that the loss of *NUC* did not affect the germination, but that the overexpression of *NUC* accelerated the germination by 24–36 h (Figure 1b). At the seedling stage (measured at 15 days after germination), the loss of *NUC* did not result in any obvious morphological differences (Figure S1b), but the overexpression of *NUC* significantly increased the hypocotyl length (Figure 1c). During the juvenile stage (measured at 40 days), we found that a loss of *NUC* did not affect the plants’ growth, while the overexpression of *NUC* resulted in the accumulation of a 1.79-fold higher whole-plant biomass and 1.49-fold higher root biomass over that of *Col* (Figure 1d,e). We also found that the overexpression lines flowered early (Figure S1c), which is consistent with a previous report [1]. These results indicate that the overexpression of *NUC* extensively promotes plant development and growth. However, the loss of function of NUC did not affect any corresponding developments, indicating that NUC is not required for these processes and suggesting that the role of NUC in plant development is complicated.

### 2.2. The Overexpression of NUC Increases the Resistance to N Deficiency of Arabidopsis

**Figure 2:**
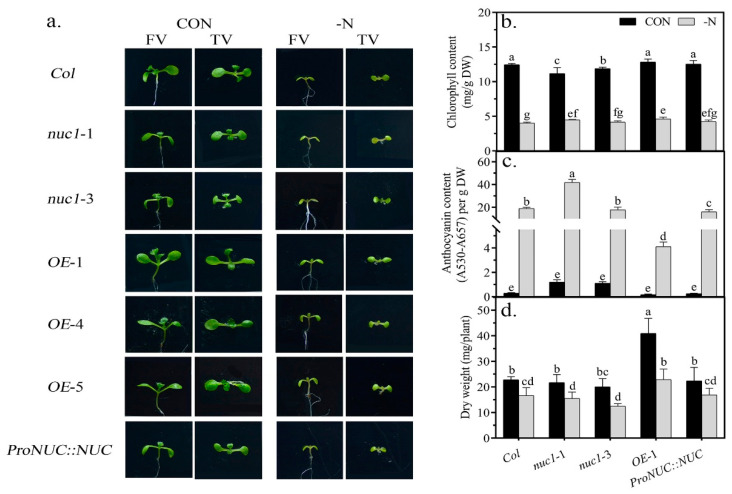
Overexpression of NUC improves plant growth under both N-sufficient and N-deficient conditions and increases resistance to N deficiency stress. (**a**): Front view (FV) and top view (TV) show the leaf phenotype of 10-day-old seedlings, in which 4-day-old normal-grown seedlings were transplanted to media with (control, CON) and without (N deficiency, -N) nitrogen for 6 days. (**b**–**d**): Chlorophyll content, anthocyanin content, and dry weight of whole plants cultured hydroponically for 45 days, in which 30-day-old seedlings were transferred to hydroponic media with (CON) and without (-N) nitrogen, respectively, for 14 days. Values are the mean ± SDs (*n* ≥ 3). Different letters indicate significant difference between treatments according to Duncan’s multiple range test at *p* < 0.05.

Considering that NUC functions in root development [5] and root development is closely related to nutrient uptake [32,33], we next tested whether NUC has any effects on the resistance to N deficiency. We transferred control-grown seedlings of the six lines mentioned above to media with (control, CON) and without (treatment, -N) nitrogen and grew them for 6 days (Figure 2a). We found that the leaves of the *NUC* overexpression lines were obviously larger than those of the other lines grown under normal growth conditions. Under N deficiency, the seedling growth of all lines was suppressed, but the growth of the *NUC* overexpression lines was much better than that of the other lines. We further used a hydroponic culture to examine the resistance to N deficiency. We precultured the *Col*, *nuc1*-1, *nuc1*-3, *OE*-1, and *ProNUC*::*NUC* lines and transferred them to hydroponic media with and without N for 14 days. We found that chlorophyll decreased (*Col* 68%, *nuc1*-1 60%, *nuc1*-3 65%, *OE*-1 64%, and *ProNUC::NUC* 66%), anthocyanin increased (*Col* 66-fold, *nuc1*-1 35-fold, *nuc1*-3 16-fold, *OE*-1 23-fold, and *ProNUC::NUC* 63-fold), and biomass decreased (*Col* 27%, *nuc1*-1 25%, *nuc1*-3 38%, *OE*-1 44%, and *ProNUC::NUC* 27%) under N-deficient conditions (Figure 2b–d). However, the adverse influences induced by N deficiency were significantly attenuated in the *OE*-1 line (Figure 2b–d). Taken together, the *NUC* overexpression lines grew better than the other lines under N deficiency. The results showed that the overexpression of *NUC* enhanced the resistance to N deficiency of *Arabidopsis*. Notably, the loss of function of NUC did not affect the resistance to N deficiency. This suggested that NUC might not directly take part in the response to N deficiency.

### 2.3. The Expression of NUC in Roots Is Not Induced by N Deficiency

**Figure 3:**
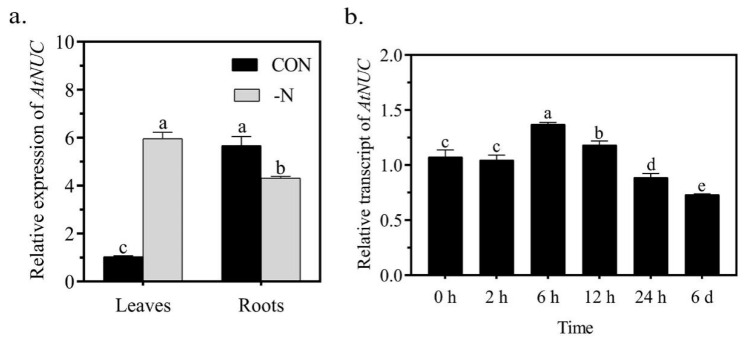
Expression of *AtNUC* in response to N deficiency. (**a**): Expression of *AtNUC* in leaves and roots under N deficiency for 6 days. (**b**): Expression of *AtNUC* in roots under N deficiency in a time course. Values are the means ± SDs (*n* = 3). Different letters indicate significant difference between treatments according to Duncan’s multiple range test at *p* < 0.05.

Higher resistance to nutrient stress could result from a better capability to capture nutrients. Such a capability is closely correlated to the root architecture. We therefore explored the mechanisms by which NUC affects the resistance to N deficiency by examining its effects on the root architecture. We first examined its expression under N deficiency. Using the FIMO tool within MEME (https://meme-suite.org/meme/tools/fimo, 9 September 2021) [34], we searched for a 2 kb promoter sequence of *NUC* and found that it contained two nitrate-responsive cis-elements (Table S2). Under normal growth conditions, the expression of *NUC* was 5.5-fold higher in the roots than in the leaves. The expression pattern supported previous reports that NUC plays a critical role in root development [3,4,5]. Under N deficiency, the expression of *NUC* was increased 6-fold in leaves and was not affected in roots (Figure 3a). In time-course measurements, the expression of *NUC* was significantly but slightly (only 28% and 10% higher than the control) induced after N deficiency treatment for 6 h and 12 h, respectively (Figure 3b). Taken together, these results indicate that the expression of *NUC* in roots was not affected by N deficiency, which further supports our aforementioned speculation that NUC might not directly take part in the response to N deficiency.

### 2.4. NUC Overexpression Constitutively Promotes Primary Root Elongation

**Figure 4:**
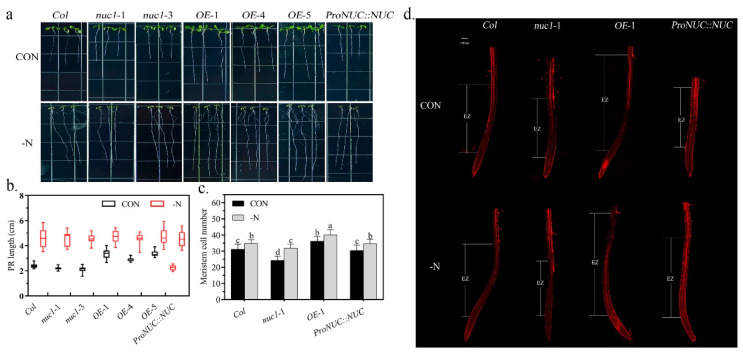
NUC overexpression promotes primary root elongation. (**a**): Root phenotype of 10-day-old seedlings, in which 4-day-old seedlings grown vertically under normal conditions were transplanted to media with (black box) and without (red box) nitrogen for 6 days. (**b**): Statistics on the primary root (PR) length of seedlings as in a (*n* ≥ 15). The horizontal bars within boxes indicate medians. The tops and bottoms of boxes indicate upper and lower quartiles, respectively. The upper and lower whiskers represent maximum and minimum, respectively. (**c**): Root cortex meristematic cell number of 5-day-old seedlings, in which 3-day-old seedlings grown vertically under normal conditions were transplanted to media with (control, CON) and without (N deficiency, -N) nitrogen for 2 days. The number of cortex cells in a single file extending from the quiescent center up to elongated cells was counted. Values are means ± SD (*n* = 10). Different letters indicate significant difference between treatments according to Duncan’s multiple range test at *p* < 0.05. (**d**): The elongation zone (EZ) in root tips. Confocal images of root tips stained with propidium iodide.

We then investigated the effects of NUC on the root architecture. We vertically grew *Col*, *nuc1*-1, *nuc1*-3, *OE*-1, *OE*-4, *OE*-5, and *ProNUC*::*NUC* lines under normal conditions for 4 days and then transplanted them to normal and N-deficient conditions for an additional 6 days (Figure 4a). Under normal growth conditions, primary roots were significantly shorter (11%) in the *nuc1*-1 and *nuc1*-3 lines and longer in the *OE*-1 (40%), *OE*-4 (21%), and *OE*-5 (39%) lines, similar to the *ProNUC*::*NUC* line than to the *Col* line (Figure 4a,b). Under N deficiency, the patterns of primary root length among the lines were basically maintained (Figure 4a,b). Given that root development is determined by the root meristem and that root length mainly comes from the elongation zone (EZ), we further examined the effects of the N deficiency on the meristem sizes and the elongation zones in these lines (Figure 4c,d). In comparison with that in the *Col* line, the meristem size and EZ length were smaller/shorter in *nuc1*-1, larger/longer in the *OE*-1 line, and the same in the *ProNUC*::*NUC* line under normal growth conditions; the size/length patterns of the meristem/EZ in all the lines were maintained under N deficiency. These results indicate that NUC positively regulated the root length, which was consistent with our previous report [5]. More importantly, however, the regulation was not affected by the N deficiency. This supports the physiological possibility that the expression of *NUC* in the roots is not affected by N deficiency (Figure 3) and means that the *NUC* overexpression constitutively promotes the primary root elongation. Notably, the longer primary roots in the *NUC*-overexpression line could contribute, at least partly, to its faster growth and better resistance to N deficiency.

### 2.5. NUC Overexpression Upregulates TGA1 and NRT2.4 under N Deficiency Conditions

**Figure 5:**
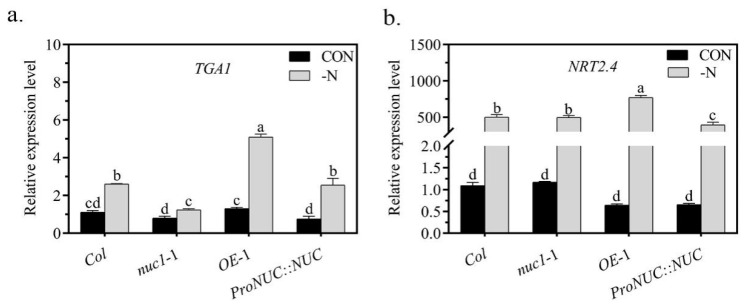
NUC regulated expression of nitrate-responsive genes. (**a**): *TGA1*. (**b**): *NRT2.4*. Values are the means ± SDs (*n* = 3). Different letters indicate significant difference between treatments according to Duncan’s multiple range test at *p* < 0.05.

Lateral roots are a part of the root architecture and are mainly responsible for increasing the surface area and searching for nutrients. They can be induced by N deficiency [35]. The involvement of the transcription factor NUC in the induced resistance to N deficiency (Figure 2) allowed us to speculate that it might regulate the nitrogen response genes responsible for the lateral root development. To further explore the mechanisms through which NUC affects the resistance to N deficiency, we screened the promoter sequences of the nitrogen metabolism genes and nitrate response genes for the NUC-specific binding sequence, the 8 bp consensus motif TTTTGTCC [1]. We searched 2 kb promoter sequences of 27 genes and found that 6 of them contained one TTTTGTCC motif (Table S3). We then examined the expression of these 6 genes in the roots of the *Col*, *nuc1*-1, *OE*-1, and *ProNUC*::*NUC* lines and found that *TGA1* and *NRT2.4* were upregulated by *NUC* under N deficiency (Figure S2 and Figure 5a,b). For example, *TGA1* expression was increased 2.3-fold by N deficiency in the *Col* line but 3.9-fold in the *OE*-1 line. Much greater increases in *TGA1* expression occurred in the *OE*-1 line. Considering that both *TGA1* and *NRT2.4* function in lateral root development [28,36], these results suggested that the inducible promotion of NUC overexpression in lateral root development might occur through the upregulation of *TGA1* and *NRT2.4* under N deficiency. In other words, *NUC* overexpression could enhance the resistance to N deficiency by upregulating *TGA1* and *NRT2.4*. Potential improvement in the lateral roots could be one of the important aspects of *NUC* overexpression to resist N deficiency.

### 2.6. NUC Overexpression Promotes the Lateral Root Development under N Deficiency

**Figure 6:**
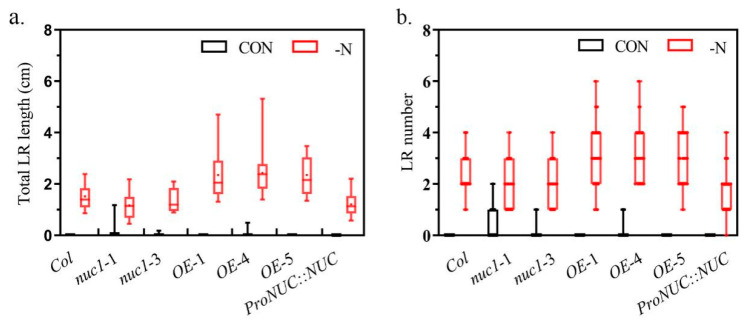
NUC overexpression promoted the development of N deficiency-induced lateral roots. (**a**,**b**): Total lateral root (LR) length and LR number per plant for the 10-day-old seedlings, in which 4-day-old seedlings grown vertically under normal conditions were transplanted to media with (black box) and without (red box) nitrogen for 6 days (*n* ≥ 15). The horizontal bars within boxes indicate medians. The tops and bottoms of boxes indicate upper and lower quartiles, respectively. The upper and lower whiskers represent the maximum and minimum, respectively.

To verify the physiological effects of the inducible upregulates *TGA1* and *NRT2.4* in the NUC overexpression lines, we compared the development of the lateral roots between the normal and N-deficient conditions. We found that these induced roots differed between the *NUC*-overexpression lines and the other lines. The length and number of lateral roots in the *OE*-1, *OE*-4, and *OE*-5 lines were greater than those in the other lines under N deficiency (Figure 6a,b). *NUC* overexpression promoted the development of N deficiency-induced lateral roots (Figure 6a,b). The results suggested that *NUC* overexpression could enable plants to reach more N sources and thus enhance their resistance to N deficiency.

We noted that *TGA1* was increased 1.5-fold by N deficiency in *nuc1*-1 (Figure 5a). These results suggest that there could be a regulatory pathway bypassing NUC and that the regulatory pathway could occur through genes, such as the transcription factor genes MGP and JKD, functioning as NUC redundantly [3]. We therefore searched the promoter sequences of the 27 nitrogen-response genes with the MGP-specific binding sequence TTGTCT [37] and found 15 genes, including *TGA1* (Table S4). Specifically, *TGA1* mediated the development of the lateral roots under N deficiency and could be regulated by MGP when NUC was absent. The evidence supports our finding that *NUC* overexpression promoted, but knockout did not weaken, the resistance to N deficiency (Figure 2).

## 3. Conclusions

In this study, we found that the transcription factor NUC had a major effect on promoting plants’ growth and a significant effect on plants’ resistance to N deficiency stress. The overexpression of NUC promoted plants’ development at the germination, seedling, juvenile, and flowering stages; it also increased the chlorophyll content, suppressed anthocyanin accumulation and increased the biomass under N deficiency. The expression of *NUC* was significantly induced in leaves but not affected in roots by N deficiency. *NUC* overexpression constitutively promoted the primary root length, and the length and number of lateral roots increased through inducible promotion. The inducible promotion on the root architecture could occur via the upregulation of *TGA1* and *NRT2.4* by NUC. Our results reveal that NUC may play very diverse roles in plants. It has roles in both plant development and plant environmental responses. It participates in a wide range of developmental processes, including germination, hypocotyl elongation, primary root elongation, lateral root induction, and flowering regulation. The properties involved can be constitutive and inducible. Our findings expand the understanding of the functions of NUC and provides some direction for its use. It should be mentioned that in most cases in this study, the absence of NUC did not result in any physiological effects. Multiple sources suggest that the phenomena could be due to its multiple functional redundancies, such as *MGP* [38,39]. Given that NUC is a transcription factor, is highly conserved in plants [1,39], and plays positive roles in plants’ development and stress resistance, it could be an excellent target gene to improve crop productivity.

## 4. Materials and Methods

### 4.1. Plant Material

*Arabidopsis thaliana* (Columbia ecotype, *Col*) *nuc1*-1 mutants (SALK_110117) were donated by Yang Xianpeng, Shandong Normal University (Jinan, China), and *nuc1*-3 mutants (SALK_124222) were ordered from the Arabidopsis Biological Resource Center (https://abrc.osu.edu, 19 October 2021). We constructed the *NUC*-overexpression transgenic plant under a *Col* background. The full-length cDNA of *NUC* was cloned into the binary vector pEGAD behind the CaMV 35S promoter. The *nuc1*-3 recovery line was rescued by *NUC* CDs subcloned into pEGAD vectors under the *NUC* promotor. The transgenic seedlings were screened by BASTA and PCR, and the third-generation transgenic seedlings were used in this study. 

### 4.2. Growth and Treatment Conditions

For sterile seedlings, sterilized seeds were sown on 1/2 Murashige and Skoog medium (MS) with 1% sucrose at pH = 5.7 for three or four days, and then seedlings were transferred to 1/2 MS medium with (control, CON) or without (treatment, -N) nitrogen for six days. The level of potassium was balanced using KCl. The seedlings were used to measure leaf and root phenotypes and gene expression levels.

For hydroponic seedings, sterilized seeds were first sown on 1/2 Murashige and Skoog medium (MS) without sucrose at pH = 5.7. After germination, plantlets were transferred to 1/4 Hoagland’s nutrient solution comprising Ca(NO_3_)_2_·4H_2_O (1 mM), KNO_3_ (5 mM), MgSO_4_ (0.5 mM), NH_4_H_2_PO_4_ (0.13 mM), NH_4_NO_3_ (0.13 mM), CaCl_2_ (1 mM), EDTA-Na_2_ (31 μM), FeSO_4_·7H_2_O (22 μM), H_3_BO_3_ (9.7 μM), MnCl_2_·4H_2_O (2.1 μM), ZnSO_4_·7H_2_O (0.3 μM), CuSO_4_·5H_2_O (0.2 μM), H_2_MoO_3_·H_2_O (0.14 μM), Co(NO_3_)_2_·6H_2_O (0.086 μM), and NH_4_NO_3_ (29 μM). When plants grew to the nine- or ten-leaf stage, they were transferred to 1/16 Hoagland’s nutrient solution with (CON) and without (-N) nitrogen for 14 days. The levels of calcium, potassium and phosphorus were balanced using KCl, CaCl_2_, and NaH_2_PO_4,_ respectively. The growth medium was refreshed every seven days. The seedlings were used for the measurement of the biomass and for the determination of the chlorophyll and anthocyanin contents.

All plants were grown in a controlled chamber at 22 °C with a relative humidity of 60% under a 12 h/12 h photoperiod with 120 μmol m^−2^ s^−1^ light illumination.

### 4.3. Measurement of Chlorophyll Contents

Chlorophyll contents were measured as described by Woodward and Bennett [40]. The pigments from leaves were extracted with 4 mL of dimethylformamide for 24 h in the dark at 4 °C, and the optical densities (OD_664_ and OD_647_) for each sample were measured. The chlorophyll content was calculated as follows: ((OD_664_ × 7.04) + (OD_647_ × 20.27)) × 4/sample dry weight (in grams) ×10^−3^ = milligram chlorophyll/gram dry weight (mg/g).

### 4.4. Measurement of Anthocyanin Contents

Anthocyanin contents were measured as previously described [41]. Leaves were ground with liquid nitrogen and placed in a large centrifuge tube with 1.5 mL of extraction buffer (methanol:HCl = 99:1) overnight at 4 °C. Then, 1 mL deionized water and 2.5 mL chloroform was added to each centrifuge tube in turn. The mixture was centrifuged at 6000 rpm for 5 min. Then, 0.5 mL supernatant was removed, and 0.5 mL buffer solution was added (methanol:HCl:H_2_O = 29:1:20). The mixed solution was collected to measure the absorbance at 530 nm and 657 nm. The relative anthocyanin concentration was calculated using the following equation: (A530–A657) per dry weight grams.

### 4.5. Histological Analyses and Microscopy

Propidium iodide (PI) staining [42] was performed as described previously and observed using a Leica STELLARIS 5 confocal laser scanning microscope.

### 4.6. Cis-Binding Site Enrichment

To search for the 2 kb promoter regions of 27 nitrate response genes in *Arabidopsis* in NCBI, the FIMO tool within the MEME package (https://meme-suite.org/meme/tools/fimo, 9 September 2021) was used to identify every occurrence of the cis-motif in the 2 kb promoter regions of 27 genes at a *p*-value < 0.001 [35].

### 4.7. RNA Extraction, Reverse Transcription, and Quantitative Real-Time PCR (qRT–PCR) Analysis

Total RNA was extracted by the HiPure Plant RNA Mini Kit (Magen, Shanghai, China) according to the manufacturer’s instructions, and cDNA with 1 μg of total RNA was synthesized using HiScript II Q RT SuperMix for qPCR (Vazyme, Nanjing, Jiangsu, China). Quantitative real-time PCR (qRT–PCR) was carried out in an ABI StepOne Real-Time PCR System (Applied Biosystems) with the EvaGreen 2× qPCR MasterMix ROX (Applied Biological Materials Inc. (abm), Viking Way, Richmond, BC, Canada). The results were normalized against the housekeeping gene tubulin, and relative quantification analysis was performed using the comparative CT method (2^−^^△△CT^) [43]. Gene expression in *Col* under CON conditions was set to 1.0. The *Arabidopsis tubulin* gene was used as an external reference. Measurement for each gene expression was based on three technical replicates and was independently performed twice. Primer sequences for each gene are listed in Table S1.

### 4.8. Statistical Analysis

The statistical analysis was performed by one-way ANOVA followed by Duncan’s multiple range tests at a significance level of *p* < 0.05 using SPSS 22. All charts were created using GraphPad Prism 8 (https://www.graphpad.com/, 4 October 2021).

## Supplementary Materials

The following are available online at https://www.mdpi.com/article/10.3390/ijms222111413/s1.

## Abbreviations

NUC	NUTCRACKER
IDD	INDETERMINATE DOMAIN
C2H2	Cys2His2 zinc-finger domain
N	Nitrogen
NUE	Nitrogen use efficiency
MS	Murashige and Skoog medium
CON	Control
-N	N-deficiency
qRT–PCR	Quantitative real-time PCR
PR	Primary root
LR	Lateral root
ER	Elongation zone

## References

[1] Seo P.J., Ryu J., Kang S.K., Park C.M. (2011). Modulation of sugar metabolism by an INDETERMINATE DOMAIN transcription factor contributes to photoperiodic flowering in Arabidopsis. Plant J..

[2] Jeong E.Y., Seo P.J., Woo J.C., Park C.M. (2015). AKIN10 delays flowering by inactivating IDD8 transcription factor through protein phosphorylation in Arabidopsis. BMC Plant Biol..

[3] Long Y., Smet W., Cruz-Ramirez A., Castelijns B., de Jonge W., Mahonen A.P., Bouchet B.P., Perez G.S., Akhmanova A., Scheres B. (2015). Arabidopsis BIRD Zinc Finger Proteins Jointly Stabilize Tissue Boundaries by Confining the Cell Fate Regulator SHORT-ROOT and Contributing to Fate Specification. Plant Cell.

[4] Cui H., Levesque M.P., Vernoux T., Jung J.W., Paquette A.J., Gallagher K.L., Wang J.Y., Blilou I., Scheres B., Benfey P.N. (2007). An evolutionarily conserved mechanism delimiting SHR movement defines a single layer of endodermis in plants. Science.

[5] Huang X., Li W., Zhang X. (2021). Flavonoid scutellarin positively regulates root length through NUTCRACKER. Plant Divers..

[6] Crawford N.M., Forde B.G. (2002). Molecular and Developmental Biology of Inorganic Nitrogen Nutrition. Arabidopsis Book.

[7] Canfield D.E., Glazer A.N., Falkowski P.G. (2010). The Evolution and Future of Earth’s Nitrogen Cycle. Science.

[8] Medici A., Krouk G. (2014). The primary nitrate response: A multifaceted signalling pathway. J. Exp. Bot..

[9] Bouguyon E., Gojon A., Nacry P. (2012). Nitrate sensing and signaling in plants. Semin. Cell Dev. Biol..

[10] Wang Y.Y., Cheng Y.H., Chen K.E., Tsay Y.F. (2018). Nitrate Transport, Signaling, and Use Efficiency. Annu. Rev. Plant Biol..

[11] Krapp A., Berthome R., Orsel M., Mercey-Boutet S., Yu A., Castaings L., Elftieh S., Major H., Renou J.-P., Daniel-Vedele F. (2011). Arabidopsis Roots and Shoots Show Distinct Temporal Adaptation Patterns toward Nitrogen Starvation. Plant Physiol..

[12] Ueda Y., Konishi M., Yanagisawa S. (2017). Molecular basis of the nitrogen response in plants. Soil Sci. Plant Nutr..

[13] Alboresi A., Gestin C., Leydecker M.T., Bedu M., Meyer C., Truong H.N. (2005). Nitrate, a signal relieving seed dormancy in Arabidopsis. Plant Cell Environ..

[14] Bellegarde F., Gojon A., Martin A. (2017). Signals and players in the transcriptional regulation of root responses by local and systemic N signaling in Arabidopsis thaliana. J. Exp. Bot..

[15] Kiba T., Krapp A. (2016). Plant Nitrogen Acquisition Under Low Availability: Regulation of Uptake and Root Architecture. Plant Cell Physiol..

[16] Olas J.J., Van Dingenen J., Abel C., Dzialo M.A., Feil R., Krapp A., Schlereth A., Wahl V. (2019). Nitrate acts at the Arabidopsis thaliana shoot apical meristem to regulate flowering time. New Phytol..

[17] Fredes I., Moreno S., Diaz F.P., Gutierrez R.A. (2019). Nitrate signaling and the control of Arabidopsis growth and development. Curr. Opin. Plant Biol..

[18] Sun X., Chen F., Yuan L., Mi G. (2020). The physiological mechanism underlying root elongation in response to nitrogen deficiency in crop plants. Planta.

[19] Meier M., Liu Y., Lay-Pruitt K.S., Takahashi H., von Wiren N. (2020). Auxin-mediated root branching is determined by the form of available nitrogen. Nat. Plants.

[20] Li J., Song X., Kong X., Wang J., Sun W., Zuo K. (2019). Natural variation of Arabidopsis thaliana root architecture in response to nitrate availability. J. Plant Nutr..

[21] Gaudinier A., Rodriguez-Medina J., Zhang L., Olson A., Liseron-Monfils C., Bagman A.M., Foret J., Abbitt S., Tang M., Li B. (2018). Transcriptional regulation of nitrogen-associated metabolism and growth. Nature.

[22] Brooks M.D., Cirrone J., Pasquino A.V., Alvarez J.M., Swift J., Mittal S., Juang C.-L., Varala K., Gutiérrez R.A., Krouk G. (2019). Network Walking charts transcriptional dynamics of nitrogen signaling by integrating validated and predicted genome-wide interactions. Nat. Commun..

[23] Gan Y., Bernreiter A., Filleur S., Abram B., Forde B.G. (2012). Overexpressing the ANR1 MADS-box gene in transgenic plants provides new insights into its role in the nitrate regulation of root development. Plant Cell. Physiol..

[24] Gan Y.B., Filleur S., Rahman A., Gotensparre S., Forde B.G. (2005). Nutritional regulation of ANR1 and other root-expressed MADS-box genes in Arabidopsis thaliana. Planta.

[25] Guan P., Ripoll J.J., Wang R., Vuong L., Bailey-Steinitz L.J., Ye D., Crawford N.M. (2017). Interacting TCP and NLP transcription factors control plant responses to nitrate availability. Proc. Natl. Acad. Sci. USA.

[26] Guan P., Wang R., Nacry P., Breton G., Kay S.A., Pruneda-Paz J.L., Davani A., Crawford N.M. (2014). Nitrate foraging by Arabidopsis roots is mediated by the transcription factor TCP20 through the systemic signaling pathway. Proc. Natl. Acad. Sci. USA.

[27] Zhong L., Chen D., Min D., Li W., Xu Z., Zhou Y., Li L., Chen M., Ma Y. (2015). AtTGA4, a bZIP transcription factor, confers drought resistance by enhancing nitrate transport and assimilation in Arabidopsis thaliana. Biochem. Biophys. Res. Commun..

[28] Alvarez J.M., Riveras E., Vidal E.A., Gras D.E., Contreras-Lopez O., Tamayo K.P., Aceituno F., Gomez I., Ruffel S., Lejay L. (2014). Systems approach identifies TGA1 and TGA4 transcription factors as important regulatory components of the nitrate response of Arabidopsis thaliana roots. Plant J..

[29] Canales J., Contreras-Lopez O., Alvarez J.M., Gutierrez R.A. (2017). Nitrate induction of root hair density is mediated by TGA1/TGA4 and CPC transcription factors in Arabidopsis thaliana. Plant J..

[30] Xu N., Wang R., Zhao L., Zhang C., Li Z., Lei Z., Liu F., Guan P., Chu Z., Crawford N.M. (2016). The Arabidopsis NRG2 Protein Mediates Nitrate Signaling and Interacts with and Regulates Key Nitrate Regulators. Plant Cell.

[31] Rubin G., Tohge T., Matsuda F., Saito K., Scheible W.-R. (2009). Members of the LBD Family of Transcription Factors Repress Anthocyanin Synthesis and Affect Additional Nitrogen Responses in Arabidopsis. Plant Cell.

[32] Forde B., Lorenzo H. (2001). The nutritional control of root development. Plant Soil.

[33] Gruber B.D., Giehl R.F.H., Friedel S., von Wiren N. (2013). Plasticity of the Arabidopsis Root System under Nutrient Deficiencies. Plant Physiol..

[34] Bailey T.L., Boden M., Buske F.A., Frith M., Grant C.E., Clementi L., Ren J., Li W.W., Noble W.S. (2009). MEME SUITE: Tools for motif discovery and searching. Nucleic Acids Res..

[35] Sun C.H., Yu J.Q., Hu D.G. (2017). Nitrate: A Crucial Signal during Lateral Roots Development. Front. Plant Sci..

[36] Kiba T., Feria-Bourrellier A.-B., Lafouge F., Lezhneva L., Boutet-Mercey S., Orsel M., Brehaut V., Miller A., Daniel-Vedele F., Sakakibara H. (2012). The Arabidopsis Nitrate Transporter NRT2.4 Plays a Double Role in Roots and Shoots of Nitrogen-Straved Plants. Plant Cell.

[37] Aoyanagi T., Ikeya S., Kobayashi A., Kozaki A. (2020). Gene Regulation via the Combination of Transcription Factors in the INDETERMINATE DOMAIN and GRAS Families. Genes.

[38] Levesque M.P., Vernoux T., Busch W., Cui H., Wang J.Y., Blilou I., Hassan H., Nakajima K., Matsumoto N., Lohmann J.U. (2006). Whole-genome analysis of the SHORT-ROOT developmental pathway in Arabidopsis. PLoS Biol..

[39] Kumar M., Le D.T., Hwang S., Seo P.J., Kim H.U. (2019). Role of the INDETERMINATE DOMAIN Genes in Plants. Int. J. Mol. Sci..

[40] Woodward A.J., Bennett I.J. (2005). The effect of salt stress and abscisic acid on proline production, chlorophyll content and growth of in vitro propagated shoots of Eucalyptus camaldulensis. Plant Cell Tissue Organ Cult..

[41] Neff M.M., Chory J. (1998). Genetic Interactions between Phytochrome A, Phytochrome B, and Cryptochrome 1 during Arabidopsis Development. Plant Physiol..

[42] Fernandez-Marcos M., Sanz L., Lewis D.R., Muday G.K., Lorenzo O. (2011). Nitric oxide causes root apical meristem defects and growth inhibition while reducing PIN-FORMED 1 (PIN1)-dependent acropetal auxin transport. Proc. Natl. Acad. Sci. USA.

[43] Yuan J.S., Reed A., Chen F., Stewart C.N. (2006). Statistical analysis of real-time PCR data. BMC Bioinform..

