# Supplementary material for: The Overexpression of NUC Promotes Development and Increases Resistance to Nitrogen Deficiency in Arabidopsis thaliana
Source: Int J Mol Sci. 2021 Oct 22;22(21):11413. doi: 10.3390/ijms222111413 (PMC8583770; doi:10.3390/ijms222111413)
Supplement: Supplementary file 1 [file ijms-22-11413-s001.zip › ijms-1397591-supplementary.pdf]

Supplemental materials

Figure S1. a, T-DNA insertion sites of two mutants (*nuc1*-1 and *nuc1*-3) [1]. b, Growth phenotypes of 15-day-old seedlings grown on normal 1/2 strength MS medium (*n* = 3). c, Side view of 32-day-old seedlings grown in soil.

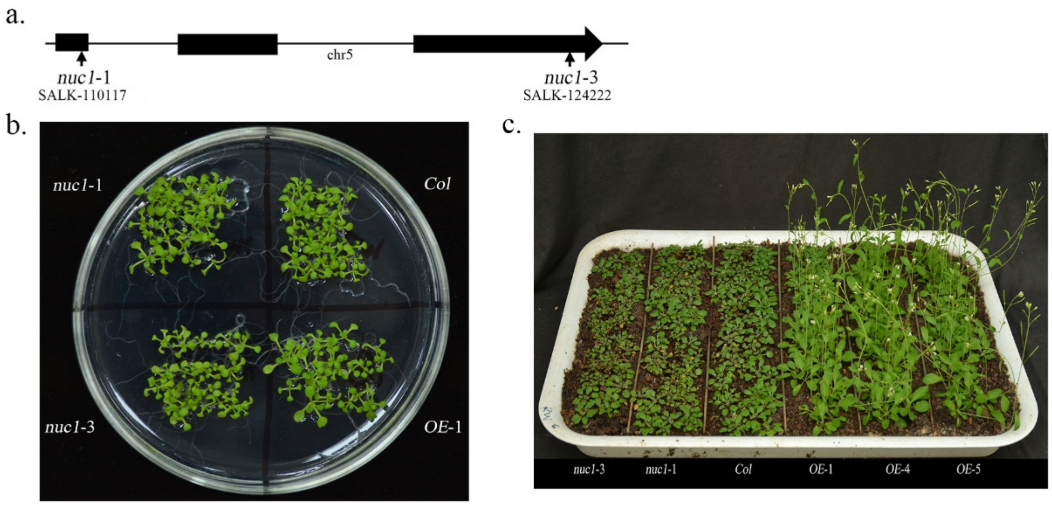

Figure S2. The expression levels of 6 genes that regulate nitrogen metabolism and nitrate response. Four-day-old seedlings grown on normal MS medium were transplanted to MS medium with (CON) or without N (−N) conditions for an additional 6 days. Then, the roots of the lines were harvested for qRT–PCR. Gene expression in *Col* under CON conditions was set to 1.0. The Arabidopsis tubulin gene was used as an external reference. Values are the mean ± SDs (*n* = 3)

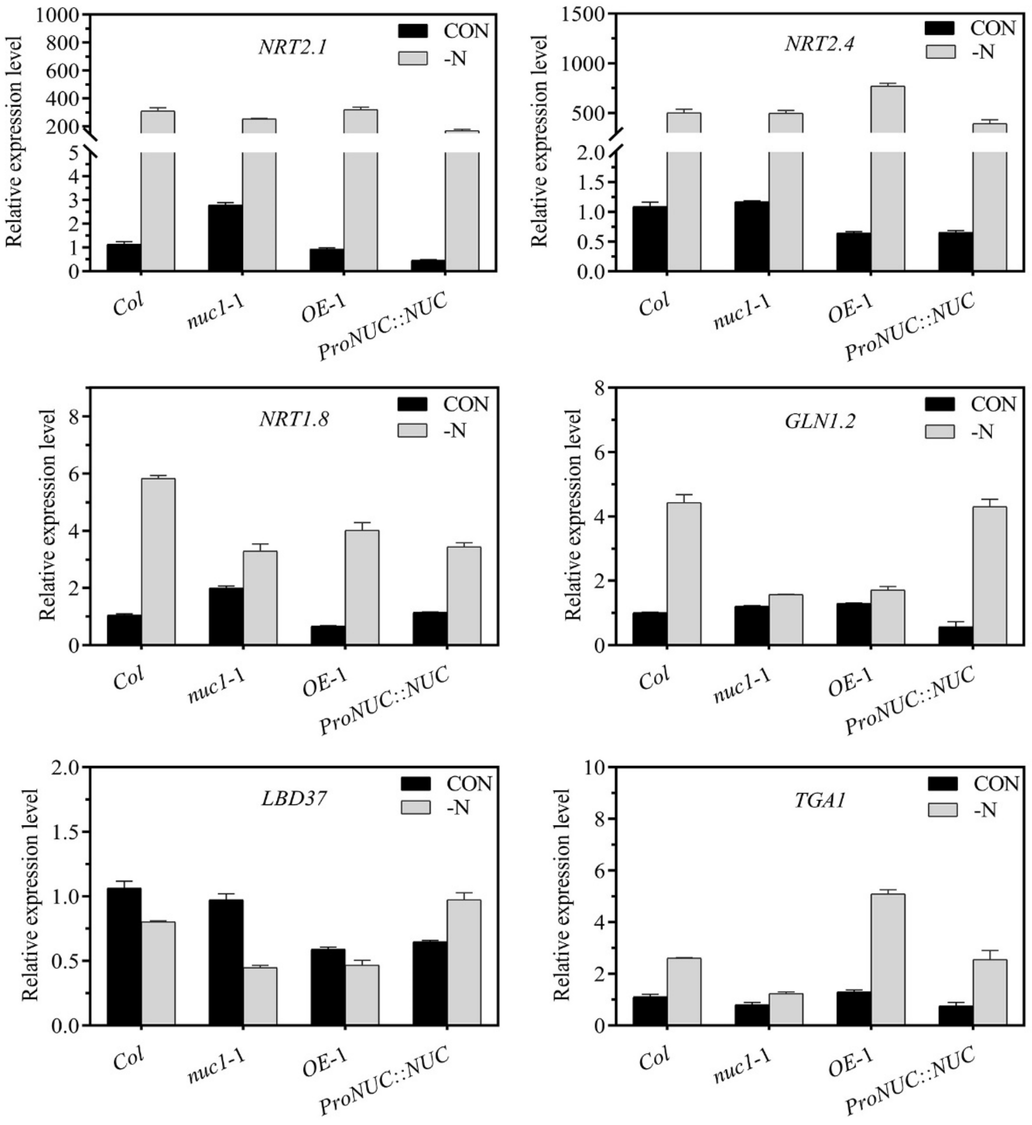

Figure S3. The lateral root phenotype of the 10-day-old seedlings, in which 4-day-old seedlings grown vertically under normal conditions were transplanted to media with (control, CON) and without (N-deficiency, −N) nitrogen for 6 days.

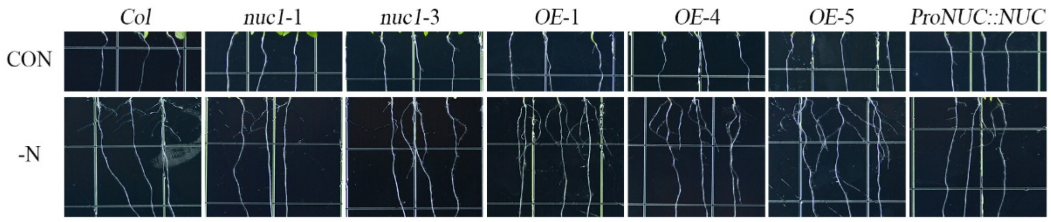

Table S1. PCR primer sequences

Gene	Forward primer	Reverse primer
Tubulin	TGTGGAGAATGCAGATGAGT	ACCATGAAGAAGTGGAGACG
NUC	TACAGAATTACCCTTGGAAACG	CAAATCCATCCATTGATAGACG
NRT2.1	TGGGAGTTGAGTTGAGCACTGA	GCCATTCCGAAACATGCTGCTA
NRT2.4	GGAAGATGAAGTGAAGTCTAC	CACGCTCCGATCTACTATT
NRT1.8	GGCTTCAGATTCTTGGATAG	AACCACAGAGTAGAGGATGG
GLN1.2	TGTGATGCTTACACTCCAG	GCCAGTTCACATCCTTCT
LBD37	AACACCTTCTTCTACACGG	TTCTCCTCCTCCGTTACC
TGA1	CCACCGAGAAGGTTTGAA	CTGACAAGCTGTCTGGTT
NUC-CDs	CGGAATTCATGACAAGTGAAGTTCTTCAA	CGGGATCCTCAAATCCATCCATTGATAGAC
NUC-P	CCTTAATTA AGAATCAGTTTAAGAGTTGTAGG	GGACCGGTGTCTTCTTCTGGGTTCTTGA

Table S2. The nitrate-responsive cis-element (NRE) of NUC

Gene	Chr	start	end	strand	score	p-val	sequence
NUC	Chr5	219	261	-	-20.9152	1.52E-05	TGAAAGAGGTAAAACAGATCCAACGGTTATGGTCACATCTCTT
NUC	Chr5	1513	1555	+	-26.697	7.42E-05	GCAAACATAGGGTGCCTATAAAGTAATCAAGGGATGTTAAATT

Table S3. NUC binding motifs in the promoters of genes

Gene	Chr	Start	End	Strand	Score	*p*-value	Function in planta
NRT2.1	Chr1	512	519	+	14.9455	1.79E-05	High-affinity nitrate transporter
NRT2.4	Chr5	579	586	-	14.9455	1.79E-05	High-affinity nitrate transporter
NRT1.8	Chr4	324	330	-	13.1818	6.51E-05	Nitrate retrieval from xylem
GLN1.2	Chr1	865	872	+	14.9455	1.79E-05	Nitrogen assimilation
LBD37	Chr5	989	996	+	14.9455	1.79E-05	Negative regulator of nitrate-related genes
TGA1	Chr5	1787	1794	+	14.9455	1.79E-05	Positive regulator of root system development

Table S4. MGP binding motifs in the promoters of genes

Gene	Chr	Start	End	Strand	Score	*p*-value	Function in planta
NRT1.2	Chr1	179	184	+	11.1394	2.89E-04	Nitrate uptake in roots and ABA-mediated inhibition of seed germination
NRT1.2	Chr1	485	490	+	11.1394	2.89E-04	
NRT1.2	Chr1	770	775	-	11.1394	2.89E-04	
NRT1.7	Chr1	242	247	-	11.1394	2.89E-04	Nitrate remobilization from old to young leaves
NRT2.1	Chr1	628	633	+	11.1394	2.89E-04	High-affinity nitrate transporter
NRT2.1	Chr1	1226	1231	-	11.1394	2.89E-04	
NRT2.2	Chr1	665	670	+	11.1394	2.89E-04	
NRT2.2	Chr1	1425	1430	-	11.1394	2.89E-04	
NRT2.5	Chr1	366	371	-	11.1394	2.89E-04	
NIA1	Chr1	444	449	-	11.1394	2.89E-04	Reduced nitrate

NIA1	Chr1	599	604	+	11.1394	2.89E-04	
NIA1	Chr1	888	893	-	11.1394	2.89E-04	
NIR	Chr2	665	670	-	11.1394	2.89E-04	
NIR	Chr2	866	871	+	11.1394	2.89E-04	
GLN1.1	Chr5	1098	1103	-	11.1394	2.89E-04	Nitrogen assimilation
GLN1.1	Chr5	1239	1244	-	11.1394	2.89E-04	
NLP6	Chr1	194	199	+	11.1394	2.89E-04	Master transcription factor in nitrate responses
NLP6	Chr1	321	326	+	11.1394	2.89E-04	
NLP6	Chr1	1666	1671	+	11.1394	2.89E-04	
NLP6	Chr1	1839	1844	+	11.1394	2.89E-04	
NLP6	Chr1	1967	1972	+	11.1394	2.89E-04	
NLP7	Chr4	1267	1272	+	11.1394	2.89E-04	
TCP20	Chr3	1714	1719	-	11.1394	2.89E-04	Positive regulator of nitrogen metabolism
LBD37	Chr5	558	563	-	11.1394	2.89E-04	Negative regulator of nitrate-related genes
LBD37	Chr5	1820	1825	+	11.1394	2.89E-04	
LBD37	Chr5	1820	1825	+	11.1394	2.89E-04	
LBD38	Chr5	1841	1846	+	11.1394	2.89E-04	
LBD39	Chr5	1264	1269	+	11.1394	2.89E-04	
TGA1	Chr5	660	665	-	11.1394	2.89E-04	Positive regulator of root system development
